# Current insights into circulating biomarkers and their potential for predicting adolescent idiopathic scoliosis progression

**DOI:** 10.3389/fcell.2026.1760636

**Published:** 2026-02-06

**Authors:** Lavinia Raimondi, Alessandra Colombini, Alberto Ruffilli, Fabrizio Perna, Stefano Negrini, Angelo Toscano, Gianluca Giavaresi

**Affiliations:** 1 Surgical Sciences and Technologies, IRCCS Istituto Ortopedico Rizzoli, Bologna, Italy; 2 Orthopaedic Biotechnology Lab., IRCCS Galeazzi S. Ambrogio Hospital, Milan, Italy; 3 1st Orthopaedic and Traumatologic Clinic, IRCCS Istituto Ortopedico Rizzoli, Bologna, Italy; 4 General Orthopaedic, IRCCS Istituto Ortopedico Rizzoli, Bologna, Italy; 5 Department of Biomedical, Surgical and Dental Sciences, University of Milan, Milan, Italy; 6 Laboratory of Evidence Based Rehabilitation, IRCCS Galeazzi S. Ambrogio Hospital, Milan, Italy

**Keywords:** adolescent idiopathic scoliosis (AIS), biomarker validation in AIS, bone metabolism markers, circulating biomarkers, disease progression, microRNAs (miRNAs) detection, pathophysiology of scoliosis

## Abstract

Adolescent Idiopathic Scoliosis (AIS) is a three-dimensional deformation of the spine with a frontal plane curvature of 10° or more, measured using Cobb method. It typically gets more severe during pre-puberty and puberty, currently exhibiting unpredictable progression. Severe disease is more prevalent in females, and progression is associated with respiratory and neuromuscular dysfunction, pain, and psychological complications. Management strategies are guided by curve severity and include observation, therapeutic exercises, bracing, and surgery. Despite advances, the cellular and molecular mechanisms driving AIS remain poorly understood. Early detection and reliable progression biomarkers are increasingly recognized as critical to prevent clinical mismanagement. This mini-review summarizes current evidence on circulating biomarkers investigated in AIS, including growth-related hormones, bone metabolism proteins, and more recently non-coding RNAs (ncRNAs) such as microRNAs. In addition, we highlight key methodological limitations and risk-of-bias concerns across existing studies, especially the reliance on single-time-point sampling, underscoring the need for longitudinal prospective cohorts with repeated biomarker measurements. Such designs are critical for capturing dynamic biological changes, distinguishing stable from progressive cases, and validating biomarker trajectories for integration into clinically meaningful prediction models for AIS progression.

## Introduction

Adolescent idiopathic scoliosis (AIS) represents the most frequent type of structural spinal deformity with an unknown cause, characterized by a frontal Cobb angle of ≥10° on radiographs, in the absence of congenital or neuromuscular conditions (Weinstein et al., 2008). AIS affects roughly 1%–4% of adolescents during the early stages of puberty (Weinstein et al., 2008). About 80% of idiopathic scoliosis cases are identified during adolescence, which spans from 10 years of age until skeletal maturity (Asher and Burton, 2006). It remains common and increasing worldwide and shows regional variation in its prevalence (Wang S. et al., 2025). A 2010 meta-analysis estimated the global prevalence of AIS with spinal curves of ≥10° at 1.34% (Fong et al., 2010). In a large longitudinal cohort study, 2.5% of subjects developed spinal curves ≥10°, while 1.4% of subjects had curves ≥20° during adolescence (Luk et al., 2010).

The prevalence of AIS varies with sex and age. It is more frequent in girls than in boys, with reported female-to-male ratios from 1.5 to 11, depending on study design (Fong et al., 2010; Ueno et al., 2011; de Souza et al., 2013). Screening is performed with the forward bend test and scoliometer, and cases are classified by age at diagnosis and curve location. Severity is defined by Cobb angle (Cheng et al., 2015), which guides clinical features and management. Mild curves (≤20°) usually cause trunk asymmetry or subtle postural changes, are often asymptomatic, and are treated with observation or therapeutic exercise. Moderate curves (21°–35°) present with trunk asymmetry, occasional back pain, and increased progression risk; bracing is typically indicated. Moderate-to-severe curves (36°–45°) add more evident postural changes, back pain, and sometimes elevated pulmonary artery pressure; bracing remains the standard therapeutic approach. Severe curves (46°–55°) are commonly associated with reduced pulmonary function, exertional dyspnea, and cosmetic deformity, and often prompt surgical consideration. Very severe curves (≥56°) can cause marked loss of lung volume, dyspnea, alveolar hypoventilation, and chronic respiratory failure, for which surgery is generally required (Negrini et al., 2018; Koumbourlis, 2006). Progression risk is highest in skeletally immature patients, particularly when the initial Cobb angle exceeds 25°, and lowest in older, taller, or post-menarche patients with smaller curves (Lee et al., 2012; Negrini et al., 2025; Parent et al., 2023; Negrini et al., 2024).

Accurately predicting progression in AIS remains a major clinical challenge. Few patients progress, yet current methods cannot reliably identify them early. Treatment decisions, including bracing and surgery, still rely largely on estimated risk of curve progression. As a result, many young patients receive excessive treatment—such as unnecessary bracing or frequent radiographs—while some progressive cases are recognised only when surgical intervention is inevitable (Weinstein et al., 2008; Sitoula et al., 2015). Prolonged follow-up and brace wear can also impair body image and quality of life. These limitations highlight an urgent need for objective, non-invasive, and early indicators of curvature progression. Ideally, predictors should: (1) be measurable in easily accessible samples (e.g., blood); (2) capture the biological processes driving scoliosis progression and the impact of therapy on these pathways; and (3) support patient stratification for personalized monitoring and treatment. Within this framework, circulating biomarkers (such as microRNAs, hormonal factors, inflammatory mediators, and peptides) are emerging as promising tools for minimally invasive, early detection of AIS progression risk.

Research on circulating biomarkers in AIS has progressed, identifying candidates associated with disease onset and progression, with potential to enhance early diagnosis, prognostic assessment, and monitoring. Clinical translation, however, remains limited by methodological heterogeneity and the scarcity of longitudinal studies validating temporal changes and clinical relevance. Because prediction depends on dynamic evaluation, longitudinal designs provide the strongest evidence of biomarker utility, whereas cross-sectional studies mainly elucidate underlying mechanisms. This mini-review summarises key cross-sectional, case-control, and longitudinal studies, outlining how each design contributes, with different inferential strength, to identifying and validating circulating biomarkers in AIS progression.

## Literature search strategy

A literature search was conducted in PubMed, Scopus, and Web of Science to identify original research on human subjects, published in English in the last 10 years (up to October 2025). Search terms included “adolescent idiopathic scoliosis,” “circulating biomarkers,” “microRNAs,” “inflammatory cytokines,” “hormonal markers,” and “extracellular vesicles.” Review articles, case reports, and conference abstracts were excluded. The search yielded 21 eligible studies. Longitudinal clinical investigations of circulating biomarkers in AIS were prioritized, as they provide insight into temporal changes and possible relations with curve progression. Preference was also given to recent articles in high-impact journals and to studies most relevant to the specific aims of this mini-review.

## Results

### Circulating biomarkers in AIS

*microRNAs* (miRNAs) are small, non-coding RNAs that regulate gene expression post-transcriptionally and are highly stable in body fluids, making them promising non-invasive biomarkers and potential therapeutic targets (Condrat et al., 2020; Huber et al., 2023). The regulation of several pathological processes by miRNAs includes bone loss, metastasis, cancer cell proliferation, and osteoblast and osteoclast differentiation (Sharma et al., 2023; Doghish et al., 2023). In AIS several studies have investigated circulating miRNAs in relation to abnormal bone phenotypes and disease progression (Zhang et al., 2018). Preclinical studies have examined miRNAs in AIS as regulators of critical molecular pathways and as potential biomarkers for disease monitoring and therapeutic guidance. Clinical studies have compared circulating miRNAs between age-matched AIS patients and controls, and stratified patients to more accurately define the multifactorial nature of the disease. Nonetheless, the translation of these markers into clinical practice remains limited by methodological variability of the studies, small sample sizes, and the absence of longitudinal validation.

miR-145-5p has been shown to disrupt osteocyte function via the Wnt/β-catenin pathway, correlating negatively with bone markers such as sclerostin, osteopontin, and osteoprotegerin (Zhang et al., 2018), while miR-96-5p and miR-224 are elevated in AIS plasma and regulate osteoblast activity, correlating with bone to certain bone quality parameters and turnover markers (P1NP and CTx). Notably, miR-96-5p shows potential as a biomarker for diagnosing AIS and may contribute to the low bone mass observed in these patients (Chen et al., 2022; Cheng et al., 2020).

Next-generation sequencing identified a six-miRNA signature (miR-122-5p, miR-671-5p, miR-223-5p, miR-1226-5p, miR-27a-5p, miR-1306-3p), with a validated four-miRNA panel achieving AUC 0.95, targeting genes involved in bone metabolism and Wnt signaling (García-Giménez et al., 2018). A composite predictive model combining clinical features (Cobb angle, Risser sign, age, menarche) with biomarkers such as miR-167 and P1NP allowed prediction of severe curve progression (>40° Cobb angle) over long-term follow-up. The model demonstrated improved predictive accuracy compared to individual parameters, achieving a high hazard ratio (HR) further validated in an independent cohort with 72.7% sensitivity and 90% specificity (Zhang et al., 2020). Additionally, circulating miRNAs carried in extracellular vesicles, particularly the miR-30 family, were elevated in severe AIS females and impaired osteoblast differentiation, suggesting an active role in disease progression (Raimondi et al., 2024). These findings allow speculation that circulating miRNAs may reflect AIS severity and curve progression, displaying sex- and age-specific patterns potentially influenced by hormonal factors such as age at menarche. miRNA sequencing identified expression profiles in severe and mild AIS patients and healthy controls. Bioinformatic analysis and validation into a larger cohort—matched for clinical characteristics except for main curve Cobb angle—revealed elevated miR-151a-3p expression with the highest diagnostic accuracy for severe AIS, associated with dysregulated bone metabolism (Wang et al., 2020). Genome-wide plasma miRNA profiling and Random Forest modeling identified a six-miRNA panel (miR-1-3p, miR-19a-3p, miR-19b-3p, miR-133b, miR-143-3p, miR-148b-3p) that accurately predicts scoliosis progression, highlighting their potential as early circulating biomarkers in AIS (Khatami et al., 2025).

Overall, these results support the use of circulating miRNAs as diagnostic biomarkers and lay the groundwork for a predictive model of AIS progression integrating validated biomarkers and clinical features.

*cell-free DNA* (ccf-DNA, nuclear (n) and mitochondrial (mt)) is considered a promising biomarker due to its biological origin, detectability in blood, and association with pathophysiological processes. Ccf-DNA fragments are released by cells undergoing apoptosis, necrosis, or through active secretion, reflecting cell turnover and tissue damage. Ccf-DNA differs in AIS patients relative to controls and associates with clinical parameters. Plasma n-ccf-DNA was decreased in AIS, while mt-ccf-DNA was largely unchanged. Sex-specific differences included higher n-DNA in male controls versus male AIS and female controls, and elevated mt-DNA in female AIS versus male AIS. Lenke type-specific patterns revealed that Lenke type 1 patients had lower ccf n-DNA levels, whereas Lenke type 5 patients had higher ccf mt-DNA levels compared with those of controls. However, no significant correlations with Cobb angle were observed (Li et al., 2019).

*Exosomes* impact bone growth by delivering functional molecules, including proteins and miRNAs, to bone marrow cells, thereby regulating the balance between bone formation and resorption (Lyu et al., 2020). Given their role in bone metabolism, exosome-derived miRNAs are emerging as promising biomarkers for AIS, useful for diagnosis and monitoring disease. Results showed that low expression of miR-27a-5p, miR-539-5p, and miR-1246 effectively differentiated AIS patients from healthy controls (Yuan et al., 2024). While these studies yield promising early results, the absence of longitudinal data limits validation.

*Proteins* represent promising potential blood biomarkers in AIS. A proteomic approach identified a differential protein expression in plasma exosomes of AIS patients, highlighting Cartilage intermediate layer protein 1 (CILP-1) and the transforming growth factor beta 1 (TGF-β1)/Smad pathway as key factors in muscle asymmetry and fibrosis, with potential implications for AIS progression. The study subdivided AIS patients into a “follow-up” group (Cobb angle <40°) and a “surgery” group (Cobb angle ≥40°) to assess progression. Exosome proteomic profiles differentiated early/milder from more advanced AIS, suggesting protein changes correlate with severity or progression. Elevated CILP-1 expression and its muscle-side asymmetry, linked to curve severity, proposed it as a potential progression biomarker. The connection between exosomal protein changes (systemic biomarker) and local muscle pathology (concave-side fibrosis/alteration) highlighted how molecular alterations may drive the structural and biomechanical evolution of the curve (Wang Q. et al., 2025).

A cross-sectional study comparing plasma dipeptidyl peptidase-4 (DPP-4) activity in 113 AIS girls and 62 age-matched controls found reduced DPP-4 levels in AIS, which further decreased with increasing curve severity. Significant differences emerged at key thresholds—becoming apparent at 30° and statistically significant above 50°—suggesting circulating DPP-4 as a potential biomarker for predicting curve progression (Normand et al., 2017). Another study demonstrated that reduced DPP-4 in AIS disrupts insulin-mediated myoblast metabolism, impairing proliferation and myogenesis, which may contribute to musculoskeletal abnormalities linked to AIS severity and progression, even though direct correlations with curve progression were not assessed (Dai et al., 2022). Pointing to an inflammatory component, the study found that specific inflammatory blood proteins—particularly beta-2 and gamma globulins—correlate with AIS severity, suggesting that inflammation may contribute to its progression and could aid in disease monitoring (Bertelè et al., 2024). A mendelian randomization study identified resistin (RETN) as a causal inflammatory cytokine associated with scoliosis, highlighting its potential role in disease pathogenesis and as a therapeutic target (Mardan et al., 2024).

*Metabolites and Peptides*. A serum metabolic profiling of AIS patients versus healthy controls identified seven differential metabolites—mainly lipids (glycerophospholipids, glycerolipids, a fatty acyl carnitine) and one glucuronic acid derivative—indicating perturbed lipid metabolism in AIS pathogenesis and serve as potential diagnostic biomarkers. Stratification by Cobb angle (mild <40°, moderate 40°–50°, severe >50°) showed no clear association between metabolite levels and curve severity (Sun et al., 2016). A separate metabolomic study identified metabolites (e.g., arginine, N-acetylaspartate, citrate) significantly associated with clinical measures—including Cobb angle—in AIS patients, using Spearman correlation. However, the analysis was cross-sectional (i.e., curve size at time of sampling) without stratifying patients by longitudinal progression. Thus, while certain metabolites correlated with curve magnitude, the study did not provide direct evidence that they predict the rate of curve progression (Xiao et al., 2021).

A recent study investigated whether circulating muscle-derived myokines could predict brace treatment outcomes in treatment-naïve AIS girls aged 10–14 years (Cobb 20°–35°, Risser 0–4). Lower baseline levels of FSTL1, apelin, fractalkine, and musclin were associated with curve progression, with FSTL1 emerging as an independent predictor of brace failure (AUC = 0.729). Given its role in bone metabolism, energy regulation, and muscle activity, reduced FSTL1 may indicate impaired muscle–bone signaling in AIS. These findings highlight skeletal muscle secreted factors as potential biomarkers for predicting bracing outcomes and guiding personalized interventions (Feng et al., 2023).

*Hormones*. Curvature often begins during periods of hormonal change, with the onset of idiopathic scoliosis frequently aligning with key hormonal shifts. Hormonal imbalances are common in scoliosis and during puberty are related to the onset and progression of AIS also through a direct influence on bone growth. Addressing circulating hormones as biomarkers may be crucial for managing curvature progression in AIS (Leboeuf et al., 2009).

Leptin and ghrelin are two key factors that regulate energy balance, growth and glucose/lipid metabolism (Schorr and Miller, 2017).

Several studies have reported serum leptin and ghrelin level disorders in AIS patients (Liang et al., 2012; Liu et al., 2012; Qiu et al., 2007; Sales de Gauzy et al., 2015; Tam et al., 2014; Wang et al., 2016). Circulating level of leptin were low (Qiu et al., 2007) and the circulating level of ghrelin were high (Sales de Gauzy et al., 2015) in AIS girls. These data have been confirmed in a more recent study (Yu et al., 2018). Interestingly, this study reported ghrelin as new quantitative indicator (levels of >6.48 ng/mL) for predicting curve progression in AIS girls in 18 months of follow-up. Since ghrelin regulates bone remodeling and promotes osteoblast activity through the CREB and Runx2 pathways (Ma et al., 2015), it may play a role in the low bone mineral density commonly observed in girls with AIS (Cheng et al., 2001). A study found a link between another adipokine (adiponectin) and osteopenia in AIS, with genetic variation possibly playing a role (Zhang et al., 2019). The authors showed that adiponectin levels were significantly higher in AIS patients with osteopenia than in those with normal bone mass and in controls. Although patients with normal bone mass showed moderately elevated levels, the difference from controls was not significant. Together with previous studies (Clark et al., 2014), these data suggest that adiponectin may play a specific role in AIS-related osteopenia. Adiponectin also increased RANKL and IL-6 expression in AIS primary cells more than in normal cells, promoting osteoclast activation, differentiation, and survival, thereby contributing to reduced bone mass. Finally, a pilot study investigated serum adipokines (leptin, adiponectin, resistin and visfatin) in a case-control study and suggested that adipokines are implicated in AIS development and/or progression (Normand et al., 2022). Even in this case, girls with AIS exhibited higher adiponectin levels and a lower leptin/adiponectin ratio compared to controls. Additionally, AIS participants with a Cobb angle greater than 25° showed elevated resistin levels relative to controls. Finally, melatonin may also contribute to AIS-related bone alterations. Studies on melatonin suggest that impaired signaling, rather than low serum levels, may influence osteoblast activity and bone growth in AIS, as circulating melatonin shows no clear correlation with Cobb angle (Brodner et al., 2000; Bagnall et al., 1996; Sadat-Ali et al., 2000; Azeddine et al., 2007).

An overview of the selected studies, including the clinical features and the methodological approaches with the main findings, is provided in Supplementary Table S1.

## Conclusions: evidence and interpretation

The unclear etiology of AIS has prompted interest in circulating biomarkers to improve early diagnosis, prognosis, and treatment monitoring. Recent studies increasingly focus on circulating biomarkers as tools to better understand and predict disease progression. Growth factors, bone metabolism proteins, myokines, and microRNAs have been linked to curve severity and progression, though most evidence is cross-sectional. When interpreted in light of the risk-of-bias assessment reported in the Supplementary Material, the available evidence highlights substantial methodological limitations that currently prevent direct clinical application of most circulating biomarkers in AIS. The analysis showed that the studies available are cross-sectional, rely on single time-point measurements, or are methodologically heterogeneous, which limits their prognostic reliability. Only a limited number of studies demonstrate a low risk of bias, and high-quality evidence. Therefore, the current lack of clinically applicable biomarkers reflects an objective limitation of the existing evidence rather than a negative or inconclusive interpretation. Among studies with low risk of bias, Yuan et al. (2024) provided robust evidence on AIS prevalence in specific regions and highlighted plasma exosome-derived microRNAs as potential diagnostic biomarkers, supporting surveillance and early-diagnosis research. Mardan et al. (2024) used two-way Mendelian randomisation to investigate the causal links between inflammatory cytokines and scoliosis, providing mechanistic insights and good-quality evidence to guide future diagnostics and treatments. Lastly, Yu et al. (2018), a good-quality and low-bias study, showed that elevated ghrelin predicts curve progression in girls with AIS, offering a reliable biomarker for early monitoring. Despite these promising findings, no single biomarker currently offers sufficient predictive accuracy, and longitudinal investigations are necessary to confirm their predictive value. Future research should aim to integrate multiple biomarkers with clinical parameters into composite predictive models capable of improving risk stratification, managing personalized treatment and thus ensuring better clinical decision-making in AIS.

## Challenges and future directions

Recent findings highlight the promise of circulating biomarkers for predicting AIS progression, but challenges remain. Heterogeneity of study designs, small sample sizes and variability in analytical methods limit comparability. Most investigations lack longitudinal follow-up, which is essential. Future Research should aim to:

1. Standardize the collection, normalization and validation of circulating biomarkers in AIS.
2. Do large, long-term studies combining molecular, hormonal and clinical data.
3. Develop and validate predictive models combining biomarkers with other data to improve risk stratification and treatment.
4. Investigate the mechanistic links between biomarkers and the pathways driving spinal deformity.

Addressing these will be crucial to transform circulating biomarker discoveries into tools capable of improving early detection, predicting progression, and guiding management in AIS.
