# Supplementary material for: Current insights into circulating biomarkers and their potential for predicting adolescent idiopathic scoliosis progression
Source: Front Cell Dev Biol. 2026 Feb 6;14:1760636. doi: 10.3389/fcell.2026.1760636 (PMC12920475; doi:10.3389/fcell.2026.1760636)
Supplement: Supplementary file 1 [file Table1.docx]

Table 1. Overview of clinical and translational studies evaluating circulating omics-based biomarkers in adolescent idiopathic scoliosis.

| **Study -omics type** | **Design** | **Population /**  **N, Follow-up /**  **Time** | **Circulating factor(s)** | **Methodology** | **Main findings** | **Clinical implications/**  **utility** | **RoB/**  **Overall quality** | **Ref.** |
| --- | --- | --- | --- | --- | --- | --- | --- | --- |
| ***microRNAs*** | | | | | | | | |
| Bone and plasma miRNA-omics with associated protein marker profiling. | Experimental translational study combining human bone biopsies, in vitro functional assays and circulating biomarker analysis. | Bone tissue from 13 AIS patients and 10 non‑AIS subjects (mean age ~15.6 ± 5.8 years); serum biomarker analysis in 74 individuals (AIS and controls); no long-term clinical follow-up. | miR‑145‑5p and its downstream pathway (β‑catenin, SOST, osteopontin, osteoprotegerin) in bone and circulation. | Gene‑wide miRNA microarray of bone tissue (Agilent) with RT‑qPCR validation; Western blot and immunohistochemistry for β‑catenin pathway proteins; co‑immunoprecipitation; Luminex multiplex assays and ELISAs for serum bone markers; correlation of circulating miR‑145 with OPG, OPN and SOST. | miR‑145‑5p was overexpressed in AIS bone and osteoblasts, impairing osteocyte function via β‑catenin signaling. Circulating miR‑145 levels correlated negatively with serum sclerostin, osteopontin and osteoprotegerin, consistent with altered bone remodeling in AIS. | Abnormal miR‑145/β‑catenin signaling may contribute to poor bone quality in AIS; circulating miR‑145 emerges as a potential biomarker and therapeutic pathway target. | Moderate/  Fair | Zhang J.  (2018) |
| miRNA-omics in bone and plasma plus imaging (DXA, HR‑pQCT). | Prospective case–control discovery/validation study, cross-sectional (single time point). | 100 adolescent girls with AIS and 50 age-matched healthy girls; subset of 4 AIS and 4 control bone biopsies for tissue analyses; no longitudinal follow-up beyond baseline imaging. | miR‑96‑5p in plasma and bone; association with AIS and low bone mass. | MiRNA microarray screening and RT‑qPCR validation in plasma and bone; HR‑pQCT and DXA to assess bone microarchitecture and BMD; logistic regression and ROC analysis to build diagnostic models including clinical variables. | miR‑96‑5p was significantly upregulated in AIS and in girls with low BMD. Plasma miR‑96‑5p alone yielded AUC ~0.67 for AIS; combining miR‑96‑5p with age, body weight and years since menarche improved AUC to ~0.75 and linked the miRNA to bone metabolic pathways. | Identifies miR‑96‑5p as a candidate diagnostic/prognostic biomarker for AIS and low bone mass that can enhance risk stratification when added to clinical factors. | Moderate/  Fair | Chen H.  (2022) |
| Circulating and cellular miRNA-omics with functional osteoblast assays. | Retrospective case–control clinical study with in vitro functional experiments; cross-sectional for biomarker measurements. | 100 girls with AIS (Cobb ~15–80°) and 52 age-matched healthy control girls; primary osteoblasts derived from AIS and control bone tissues; no longitudinal follow-up. | Circulating miR‑96, miR‑224 and miR‑605 and their effects on osteoblast differentiation and function. | Plasma miRNAs quantified by RT‑qPCR; correlations with bone quality parameters and bone turnover markers (CTX, P1NP); gain- and loss-of-function experiments in patient-derived primary osteoblasts assessing ALP activity, mineralization (Alizarin Red S), proliferation and osteogenic marker expression. | Plasma miR‑96 and miR‑224 were significantly higher in AIS than controls and correlated with indices of reduced bone quality and altered turnover. In vitro, dysregulated miR‑96/224/605 impaired osteoblast differentiation, supporting a functional role in AIS bone pathology. | Suggests these miRNAs as potential circulating biomarkers and mechanistic mediators of osteoblast dysfunction in AIS, although they were not validated in external cohorts. | Moderate/  Fair | Cheng JCY.  (2020) |
| Plasma miRNA-omics (NGS discovery plus RT‑qPCR validation). | Prospective two-phase case–control study (discovery and independent validation), cross-sectional. | Screening cohort: 17 AIS patients and 10 healthy controls. Validation cohort: 30 AIS and 13 controls; single blood sampling; no follow-up. | Circulating plasma miRNAs forming a diagnostic signature (“osteo‑miRs”). | Next-generation sequencing of plasma miRNAs in the screening cohort; RT‑qPCR validation in the larger cohort; random forest classification; ROC analysis; in silico pathway enrichment of dysregulated miRNAs. | Identified a 4‑miRNA plasma signature (miR‑122‑5p, miR‑27a‑5p, miR‑223‑3p, miR‑1306‑3p) that distinguished AIS from controls with AUC ~0.95, sensitivity ~93% and specificity ~73%. | Supports the feasibility of radiation-free molecular diagnosis and early screening of AIS using a circulating miRNA panel. | Moderate/  Fair | García-Giménez JL.  (2018) |
| Targeted circulating miRNA (miR‑145) and serum protein biomarkers (P1NP, CTX) integrated with clinical risk modeling. | Two-phase prospective longitudinal cohort study (derivation and independent validation). | 171 Chinese girls with AIS: 120 in the derivation cohort and 51 in the validation cohort; mean baseline Cobb ~24°; followed ≥6 years (mean ~6.3 years) until skeletal maturity to determine progression to severe curve (Cobb >40°). | Composite of baseline clinical parameters and circulating biomarkers: maximum Cobb angle, menarche status, body weight, Risser sign, plasma miR‑145 and serum bone turnover markers (P1NP, CTX). | Baseline clinical and radiographic assessment; serum P1NP and CTX measured on a Roche Elecsys platform; plasma miR‑145 isolated with miRNeasy and quantified by TaqMan Advanced miRNA assays; age‑adjusted correlations; multivariable logistic regression to derive a composite risk score; ROC/AUC analysis and Cox proportional hazards modeling; application of a pre‑specified risk cut‑off (0.2) in an independent validation cohort. | The composite model combining clinical variables with miR‑145 and P1NP predicted progression to severe AIS with AUC ~0.93 and hazard ratio ~28 for high‑ versus low‑risk groups. In the validation cohort, the model achieved ~73% sensitivity and 90% specificity at the predefined cut-off. | Demonstrates that integrating circulating biomarkers with clinical factors enables accurate early prognostication of AIS progression and can inform personalized follow‑up and treatment decisions. | Low/  Good | Zhang J.  (2020) |
| EV miRNA-omics and proteomics with functional MSC assays. | Monocentric observational clinical “proof‑of‑concept” study with in vitro experiments; cross-sectional. | 20 predominantly female AIS patients (moderate and severe curves) and 10 healthy controls; single time point; no progression follow-up reported. | Differentially expressed circulating miRNAs (notably miR‑30 family members) and extracellular vesicle (EV)-derived proteins; impact of AIS‑EVs on mesenchymal stem cells (MSCs). | Plasma EV isolation and characterization; microfluidic card–based miRNA qPCR profiling; MS-based proteomics of EVs from severe female AIS; functional assays exposing human MSCs to AIS‑EVs to assess osteogenic differentiation and COL1A1 expression. | Several miR‑30 family miRNAs (e.g., miR‑30a‑3p, miR‑30a‑5p, miR‑30e‑3p, miR‑30d) were upregulated specifically in females with severe AIS. Proteomics of severe female AIS‑EVs revealed dysregulated proteins (e.g., SAA1, CFL1) linked to bone/orthopaedic disease. AIS‑EVs reduced MSC osteogenic differentiation and altered COL1A1 expression. | Suggests circulating EV‑miRNAs and proteins as candidate biomarkers of severe female AIS and implicates EVs in impaired bone formation. | Moderate/  Fair | Raimondi L.  (2024) |
| Plasma miRNA-omics combined with epigenetic and functional cell assays. | Two-phase case–control study (discovery sequencing plus larger validation), cross-sectional. | Discovery cohort: plasma from a small group of AIS patients stratified by severity and healthy controls. Validation cohort: 50 AIS patients (including severe and mild cases) and 40 healthy controls; no longitudinal follow-up. | Circulating miR‑151a‑3p and its interaction with GREM1 in relation to bone homeostasis and curve severity. | Small RNA sequencing of plasma to identify dysregulated miRNAs; RT‑qPCR validation; luciferase reporter assays to confirm miR‑151a‑3p targeting of GREM1; methylation qPCR and functional osteoblast assays assessing mineralization and gene expression. | miR‑151a‑3p was significantly overexpressed in severe AIS compared with mild AIS and controls. Overexpression of miR‑151a‑3p suppressed GREM1 and impaired mineralization in vitro. Plasma miR‑151a‑3p predicted severe AIS with ROC AUC ~0.89. | Suggests miR‑151a‑3p as a circulating biomarker of curve severity and a potential therapeutic target via modulation of GREM1 and bone formation. | Moderate/  Fair | Wang Y.  (2020) |
| Plasma miRNA-omics with machine‑learning bioinformatics. | Prospective cross-sectional biomarker study nested within a longitudinal AIS cohort; discovery and two independent validation cohorts; follow-up for curve progression until skeletal maturity (~3–6 years). | Discovery cohort: 34 AIS progressors (Cobb ≥45° at follow-up), 35 non‑progressors (including moderate progressors) and 14 healthy controls. Validation cohort 1: 6 progressors, 6 non‑progressors and 6 controls. Validation cohort 2: 15 progressors, 20 non‑progressors (including moderate progressors) and 10 controls. | Genome-wide circulating miRNAs predictive of curve progression (6‑miRNA prognostic panel). | Agilent whole-genome miRNA microarray profiling in plasma; RT‑qPCR validation; random forest machine-learning models to classify progression status; pathway and network analyses of deregulated miRNAs. | Identified 15 upregulated miRNAs in progressors. A 6‑miRNA panel (miR‑1‑3p, miR‑19a‑3p, miR‑19b‑3p, miR‑133b, miR‑143‑3p, miR‑148b‑3p) accurately predicted severe curve progression, achieving near‑perfect AUC, sensitivity and specificity in internal test sets. | Provides a proof‑of‑concept circulating miRNA panel and ML-based model for early prognostic risk stratification and personalized management of AIS. | Moderate/  Fair | Khatami N.  (2025) |
| ***Cell-free DNA*** | | | | | | | | |
| Circulating cfDNA genomics (nuclear and mitochondrial qPCR). | Cross-sectional case–control study. | 69 adolescents with AIS and 21 age-matched healthy controls; single time point; no longitudinal follow-up. | Circulating cell-free nuclear DNA (ccf n‑DNA: GAPDH, ACTB) and mitochondrial DNA (ccf mt‑DNA) as potential biomarkers. | Plasma DNA extracted and quantified by qPCR for nuclear and mitochondrial targets; group comparisons; correlations with sex, Lenke curve type and Cobb angle; ROC analysis for diagnostic performance. | ccf n‑DNA levels were significantly lower in AIS than in controls, whereas overall ccf mt‑DNA was not different but was higher in female than male AIS. Lenke type 1 curves showed lower ccf n‑DNA and Lenke type 5 higher ccf mt‑DNA; ROC curves indicated limited diagnostic accuracy. | Suggests cfDNA alterations may reflect AIS pathophysiology, but current performance is insufficient for stand‑alone diagnosis or prognosis. | Moderate/  Fair | Li J.  (2019) |
| ***EXOSOMES*** | | | | | | | | |
| Plasma exosomal miRNA-omics with machine‑learning analysis. | Nested case–control study within a large prospective AIS cohort; cross-sectional for miRNA profiling. | Exploration cohort: plasma exosomes from 6 severe AIS patients and 6 healthy controls. Validation cohort: 23 severe AIS and 23 matched controls. The underlying epidemiologic study included >84,000 screened individuals; progression follow-up, but miRNA profiling at a single time point. | Plasma exosome-derived miRNAs associated with severe AIS and curve progression. | High-throughput small RNA sequencing (Illumina TruSeq) in the discovery set; differential expression analysis; RT‑qPCR validation of selected miRNAs; random forest modeling to assess diagnostic and prognostic performance of miRNA panels. | Sequencing revealed 56 upregulated and 153 downregulated exosomal miRNAs in severe AIS versus controls. Validation confirmed that miR‑27a‑5p, miR‑539‑5p and miR‑1246 have significant diagnostic value, and exosomal miRNA panels achieved high AUCs for discriminating severe AIS. | Provides a circulating exosomal miRNA panel with potential for prognostic stratification and support for clinical decision-making regarding risk of curve progression. | Low-Moderate/  Good | Yuan P.  (2024) |
| Exosome proteomics integrated with imaging and targeted validation assays. | Cross-sectional multi-cohort case–control study with proteomic discovery and ELISA validation. | Discovery: 21 AIS girls (11 under follow-up and 10 scheduled for surgery) and 20 healthy controls. Additional validation cohorts included ~35 AIS patients and ~37 controls; no explicit longitudinal follow-up. | Plasma exosome proteins, particularly extracellular matrix and fibrosis-related markers (e.g., CILP‑1, TGF‑β1, collagens, actins, myosins). | Exosome isolation and characterization by nanoparticle tracking analysis; LC–MS/MS proteomics with bioinformatic pathway analysis; validation of selected differentially expressed proteins by ELISA, RT‑qPCR, Western blot and immunohistochemistry; correlation with muscle MRI findings. | Differentially expressed exosomal proteins in AIS were enriched in pathways of extracellular matrix remodeling, inflammation, oxidative stress and muscle fibrosis. CILP‑1 and several structural proteins were elevated, including higher CILP‑1 on the concave side of the curve. | Positions exosomal proteins such as CILP‑1 as candidate diagnostic/prognostic biomarkers of paraspinal muscle fibrosis and AIS pathophysiology. | Moderate/  Fair | Wang Q.  (2025) |
| ***PROTEINS*** | | | | | | | | |
| Targeted proteomics/enzymatic assays with ex vivo cell biology. | Cross-sectional case–control study combined with ex vivo cellular experiments. | 113 AIS patients and 62 healthy controls; bone and muscle tissues from surgery used for mechanistic assays; no longitudinal follow-up. | Plasma DPP‑4 activity and its regulation in osteoblasts and related cells. | Plasma DPP‑4 enzymatic activity measured by specific protease assays; DPP‑4 protein and mRNA assessed by Western blot and qPCR; functional experiments in osteoblasts examining responses to glucose/insulin and effects on bone markers. | Plasma DPP‑4 activity was ~14% lower in AIS than controls, with the greatest reduction in girls with Cobb angles >50°. Experimental data suggested altered DPP‑4 signaling affecting bone remodeling and energy homeostasis. | Proposes DPP‑4 as a biomarker candidate and mechanistic link between metabolism and bone in AIS, though not validated as a diagnostic test. | Moderate/  Fair | Normand E.  (2017) |
| Targeted proteomics (enzyme activity/protein expression) and gene expression profiling. | Cross-sectional case–control clinical study with complementary in vitro cellular experiments. | 80 AIS girls and 50 healthy controls for serum and metabolic measurements; skeletal muscle biopsies from AIS and control subjects for mechanistic studies; no longitudinal follow-up. | DPP‑4 serum level and activity; insulin sensitivity; muscle gene expression and myogenesis. | Serum DPP‑4 measured by ELISA; DPP‑4 protein and mRNA expression in muscle assessed by Western blot and qPCR; in vitro myoblast experiments (AIS or low DPP‑4 models) evaluating insulin response, differentiation and myogenic marker expression. | AIS patients had markedly reduced serum and muscle DPP‑4 and a blunted DPP‑4 response to insulin. Impaired DPP‑4 signaling was associated with defective muscle differentiation and features of insulin resistance. | Provides mechanistic insight into muscle–insulin resistance pathways involving DPP‑4 in AIS, rather than a ready-to-use diagnostic test. | Moderate/  Fair | Dai Z.  (2022) |
| Serum proteomics (globulin fractions and immune markers). | Cross-sectional correlational clinical study. | 34 AIS patients (mean age ~14 years; 30 girls and 4 boys); single blood sampling and radiographic assessment; no follow-up. | Systemic inflammatory blood proteins (serum globulin fractions) and white blood cell subpopulations in relation to curve severity. | Serum protein electrophoresis to quantify alpha‑2, beta‑1, beta‑2 and gamma globulins; measurement of immunoglobulins and WBC differential counts; calculation of a composite “Scoliosis Score”; correlation and multivariable regression analyses with Cobb angle and Scoliosis Score. | Higher beta‑2 globulin and lower gamma globulin levels correlated with larger Cobb angles. The Scoliosis Score correlated with globulin fractions and lymphocyte/neutrophil proportions, indicating that low‑grade systemic inflammation is associated with greater curve severity. | Suggests inflammatory protein patterns as potential markers of AIS severity and as candidates for longitudinal monitoring, although they are not diagnostic on their own. | Moderate/  Fair | Bertelè L.  (2024) |
| GWAS-based genomics and Mendelian randomization (causal inference of cytokine–scoliosis relationships). | Two-sample Mendelian randomization study based on summary statistics from genome-wide association studies (GWAS); no clinical follow-up. | Exposure GWAS: 44 circulating inflammatory cytokines in 8,293 Finnish individuals. Outcome GWAS: scoliosis in 165,850 European-descent participants (1,168 cases, 164,682 controls). | Genetically proxied circulating inflammatory cytokine levels (e.g., resistin/RETN, IL‑6, IL‑17, CTACK, VEGF, TNF‑β) in causal relation to scoliosis risk. | Selection of SNPs strongly associated with each cytokine as instrumental variables; primary inverse-variance weighted Mendelian randomization; sensitivity analyses (MR‑Egger, weighted median and mode); tests for heterogeneity (Q statistics) and pleiotropy (Egger intercept, MR‑PRESSO); leave‑one‑out analyses. | Genetic liability to higher resistin (RETN) levels was causally associated with increased risk of scoliosis (IVW OR ~1.34; 95% CI ~1.04–1.74). No other cytokine showed a significant causal effect. Reverse MR provided no evidence that scoliosis causally alters cytokine levels. | Provides evidence that resistin is a causal inflammatory factor for scoliosis susceptibility, highlighting inflammation and RETN signaling as potential targets for prevention or treatment. | Low/  Good | Mardan M.  (2024) |
| ***METABOLITES AND PEPTIDES*** | | | | | | | | |
| Serum metabolomics (lipid-focused). | Two-stage case–control metabolomics study (discovery plus replication), cross-sectional. | Discovery set: 30 AIS patients and 31 healthy controls. Replication set: 31 AIS and 44 controls; single serum sampling; no follow-up. | Serum metabolite profile (lipidomic/metabolomic signature) associated with AIS. | Untargeted metabolomics using UPLC–QTOF–MS; unsupervised (PCA) and supervised (PLS‑DA, OPLS‑DA) analyses to identify discriminant metabolites; replication of candidate metabolites in an independent cohort; ROC analysis for diagnostic performance. | Seven serum metabolites were significantly altered in AIS versus controls and replicated in the validation set. A subset panel achieved high diagnostic accuracy (AUC ~0.95) for distinguishing AIS from controls. | Provides a promising serum metabolite panel for early, non‑invasive molecular screening of AIS, pending external validation. | Moderate/  Fair | Sun ZJ.  (2016) |
| Plasma metabolomics. | Cross-sectional case–control metabolomics study. | 16 adolescents with AIS and 12 healthy controls; single plasma sampling; no follow-up. | Plasma metabolite profile as exploratory diagnostic biomarker for AIS. | Non-targeted LC–MS/MS metabolomics; multivariate statistical analysis to identify differential metabolites; correlation of altered metabolites with clinical indices (body weight, BMI, BMD, Cobb angle). | Seventy‑two plasma metabolites were significantly altered in AIS, mainly involving TCA cycle, amino acid and energy metabolism pathways. Metabolites such as oxoglutarate, L‑arginine and citrate correlated with clinical indices and showed potential diagnostic value. | Reveals broad metabolic remodeling in AIS and provides candidate plasma metabolites for future validation as diagnostic or monitoring biomarkers. | Moderate/  Fair | Xiao L.  (2021) |
| Myokines profiling | Retrospective case-control study. | 117 AIS girls; followed until brace weaning or surgery | Baseline 8 circulating myokines, especially follistatin-like 1 (FSTL1), apelin, fractalkine (CX3CL1), musclin | Multiplex assay of baseline serum; logistic regression analysis. | Failure group showed significantly lower baseline levels of FSTL1, apelin, fractalkine, and musclin vs success group; FSTL1 was an independent predictor of brace success (AUC 0.729; adjusted OR 10.46); combining FSTL1 with Risser sign improved predictive performance (AUC 0.773) | Serum FSTL1 may serve as a prognostic biomarker to stratify risk of brace failure in girls with AIS, potentially guiding frequency of follow-up, imaging, and treatment intensification or exercise prescriptions | Moderate/  Fair | Feng Z.  (2023) |
| ***HORMONES*** | | | | | | | | |
| Serum peptide hormone profiling (proteomics). | Prospective observational case–control study analyzed cross-sectionally at recruitment. | 120 AIS girls (73 aged 10–13 years, 47 aged 14–17 years) and ~80 age‑comparable healthy controls; no long‑term follow-up. | Serum leptin concentration and its relation to BMI, BMD and curve severity. | Leptin measured by ELISA; comparisons between AIS and controls using non-parametric tests and ANCOVA; within AIS, correlations with anthropometry, BMC/BMD, Cobb angle and menarche; subgroup analyses by curve magnitude. | AIS girls had significantly lower leptin levels than controls, paralleling lower BMI and BMD. Leptin correlated with growth and bone mass parameters but not consistently with curve magnitude. | Supports a mechanistic role of leptin and energy balance in AIS pathogenesis rather than use of leptin as a stand‑alone diagnostic biomarker. | Moderate/  Fair | Qiu Y.  (2007) |
| Peptidomics/hormonal biomarker profiling. | Prospective observational cohort study with case–control comparison. | 105 untreated AIS girls and 40 age-matched non‑AIS girls; baseline blood sampling and clinical assessment; follow-up up to 18 months to monitor curve progression. | Serum leptin and ghrelin levels as predictors of AIS presence and progression. | ELISA quantification of ghrelin and leptin; correlations with age, anthropometry, Cobb angle and Risser sign; multivariate logistic regression to identify independent predictors; ROC analysis to evaluate prognostic performance. | AIS girls had lower leptin and higher ghrelin than controls. Among AIS patients, higher baseline ghrelin (≥7.30 ng/mL), premenarche status, initial Cobb ≥23° and low Risser (0–2) independently predicted curve progression. Ghrelin >6.48 ng/mL yielded AUC ~0.74 with ~70% sensitivity and 72% specificity. | Indicates ghrelin as a promising prognostic biomarker for AIS progression that could be incorporated into multivariate risk models for clinical management. | Low/  Good | Yu HG.  (2018) |
| Epigenomics/transcriptomics/SNP analysis integrated with serum proteomics and bone imaging. | Observational translational multi-cohort case–control and experimental study. | Baseline clinical/biomarker cohort: 92 AIS patients (subgrouped into osteopenia vs normal bone mass) and 35 age-matched controls. Large genetic cohort: 563 AIS and 281 controls from multiple centers; single time point for circulating markers; no longitudinal follow-up. | Circulating adiponectin, RANKL/OPG and IL‑6; ADIPOQ and related gene variants; bone mass phenotypes. | Serum adiponectin, RANKL/OPG and IL‑6 quantified by ELISA; BMD assessed by DXA and micro‑CT (in models); SNP genotyping of ADIPOQ and other pathway genes; qPCR and Western blot in bone cells; functional experiments in osteoblast/osteoclast systems; correlations between genotypes, adipokines and bone status. | AIS patients with osteopenia had higher adiponectin and lower BMI than AIS with normal bone mass and controls. Specific ADIPOQ variants were associated with increased adiponectin and lower BMD. Altered RANKL/OPG/IL‑6 profiles supported a role for adiponectin-driven high bone turnover in AIS osteopenia. | Identifies high circulating adiponectin, driven in part by ADIPOQ variants, as a biomarker and mechanistic contributor to osteopenia in AIS, suggesting potential for risk stratification and precision therapies targeting adiponectin signaling. | Moderate/  Fair | Zhang HQ.  (2019) |
| Multiplex adipokine/protein profiling integrated with DXA-based imaging. | Cross-sectional pilot case–control study. | 21 AIS girls and 19 healthy control girls; single fasting blood draw; no follow-up. | Serum adipokines (leptin, adiponectin, resistin, visfatin), incretins and leptin/adiponectin ratio in relation to BMD and nutritional status. | Fasting blood analyzed using a multiplex adipokine panel and ELISAs; anthropometry, dietary intake, physical activity and BMD by DXA; subgroup analyses of AIS patients stratified by Cobb angle severity. | AIS girls were leaner with lower BMD, higher adiponectin and a lower leptin/adiponectin ratio than controls. Within AIS, those with Cobb >25° had higher adiponectin and resistin than controls, while leptin and visfatin did not differ. | Supports a role for altered adipokine profiles and energy metabolism in AIS and provides pilot biomarker data, though not yet suitable for clinical screening. | Moderate/  Fair | Normand E.  (2022) |

**Abbreviations**: ADIPOQ, adiponectin gene; AIS, adolescent idiopathic scoliosis; ALP, alkaline phosphatase; ARS, Alizarin Red S; AUC, area under the receiver operating characteristic curve; BMC, bone mineral content; BMD, bone mineral density; BMI, body mass index; ccf, circulating cell-free; cfDNA, cell-free DNA; CTX, C-terminal telopeptide of type I collagen; CX3CL1, C-X3-C motif chemokine ligand 1; DPP‑4, dipeptidyl peptidase‑4; DXA, dual-energy X‑ray absorptiometry; ECM, extracellular matrix; ELISA, enzyme-linked immunosorbent assay; EV, extracellular vesicle; FSTL1, follistatin-like 1; GREM1, gremlin 1; GWAS, genome-wide association study; HC, healthy controls; HR, hazard ratio; HR‑pQCT, high-resolution peripheral quantitative computed tomography; IL‑6, interleukin‑6; IPA, Ingenuity Pathway Analysis; IVW, inverse-variance weighted; LC‑MS/MS, liquid chromatography–tandem mass spectrometry; LDH, lactate dehydrogenase; microCT, micro–computed tomography; miRNA, microRNA; ML, machine learning; MRI, magnetic resonance imaging; MR, Mendelian randomization; MSC, mesenchymal stem cell; mt‑DNA, mitochondrial DNA; n‑DNA, nuclear DNA; NGS, next-generation sequencing; OPG, osteoprotegerin; OPN, osteopontin; OPLS‑DA, orthogonal projections to latent structures discriminant analysis; OR, odds ratio; PCA, principal component analysis; P1NP, procollagen type 1 N‑terminal propeptide; PLS‑DA, partial least squares discriminant analysis; qPCR, quantitative polymerase chain reaction; RANKL, receptor activator of nuclear factor κB ligand; RETN, resistin; RFM, random forest model; RoB, risk of bias; ROC, receiver operating characteristic; RT‑qPCR, reverse-transcription quantitative polymerase chain reaction; SOST, sclerostin; SNP, single-nucleotide polymorphism; TNF‑β, tumor necrosis factor beta; UPLC–QTOF‑MS, ultra-performance liquid chromatography–quadrupole time-of-flight mass spectrometry; VEGF, vascular endothelial growth factor; WBC, white blood cell.
