# Supplementary material for: Current insights into circulating biomarkers and their potential for predicting adolescent idiopathic scoliosis progression
Source: Front Cell Dev Biol. 2026 Feb 6;14:1760636. doi: 10.3389/fcell.2026.1760636 (PMC12920475; doi:10.3389/fcell.2026.1760636)
Supplement: Supplementary file 2 [file DataSheet1.docx]

***RISK OF BIAS ANALYSIS***

Risk of bias in each case‑control study was assessed using the National Institutes of Health (NIH) Quality Assessment Tool for Case‑Control Studies (National Heart 2021). This 13‑item tool evaluates study design, selection of cases and controls, exposure and outcome assessment, control of confounding, and statistical analysis. Each item was rated as “Yes,” “Partial,” “No,” or “Not reported/applicable” according to NIH guidance. Two reviewers (AC and LR) independently scored all studies, and disagreements were resolved by consensus with a third reviewer (GG). Risk‑of‑bias results were summarized in a traffic‑light heatmap and an overall pie chart showing the proportions of studies with low, moderate, or high risk of bias, thereby enabling transparent appraisal of the available evidence.

***RISK OF BIAS ASSESSMENT***

All studies clearly specified their objectives, the study population, and case/control definitions (Supplementary Figure 1A). However, several key domains were frequently judged at high or unclear risk of bias, particularly sample size justification, assessment of temporality, blinding of exposure assessors, and statistical control of confounding. Formal sample size calculations and explicit demonstration that exposure preceded outcome were rare. Blinding procedures were often absent or poorly described. Only a minority of studies applied multivariable adjustment, while most offered limited or no control for confounders, reducing confidence in effect estimates.

The overall risk-of-bias profile shown in Supplementary Figure 1B was consistent with these item-level findings: most studies were judged at moderate risk, a minority at low or low–moderate risk, and none at high risk. This pattern underscores the need for greater methodological rigor, particularly in sample-size justification, assessment of temporality, control of confounding, and blinding.

Studies rated as low or low–moderate risk (Yuan et al. 2024; Mardan et al. 2024; Yu et al. 2018) shared several strengths: clearly defined aims, detailed descriptions of populations and settings, and strict inclusion/exclusion criteria. Controls were carefully matched to cases or drawn from the same source population, and exposure and outcome measures relied on validated assays. Temporality was ensured through prospective designs or, in Mendelian randomization, by genetic instruments. Confounding was addressed with multivariable modelling or design-based methods, although sample-size calculations and blinding procedures remained incompletely reported.


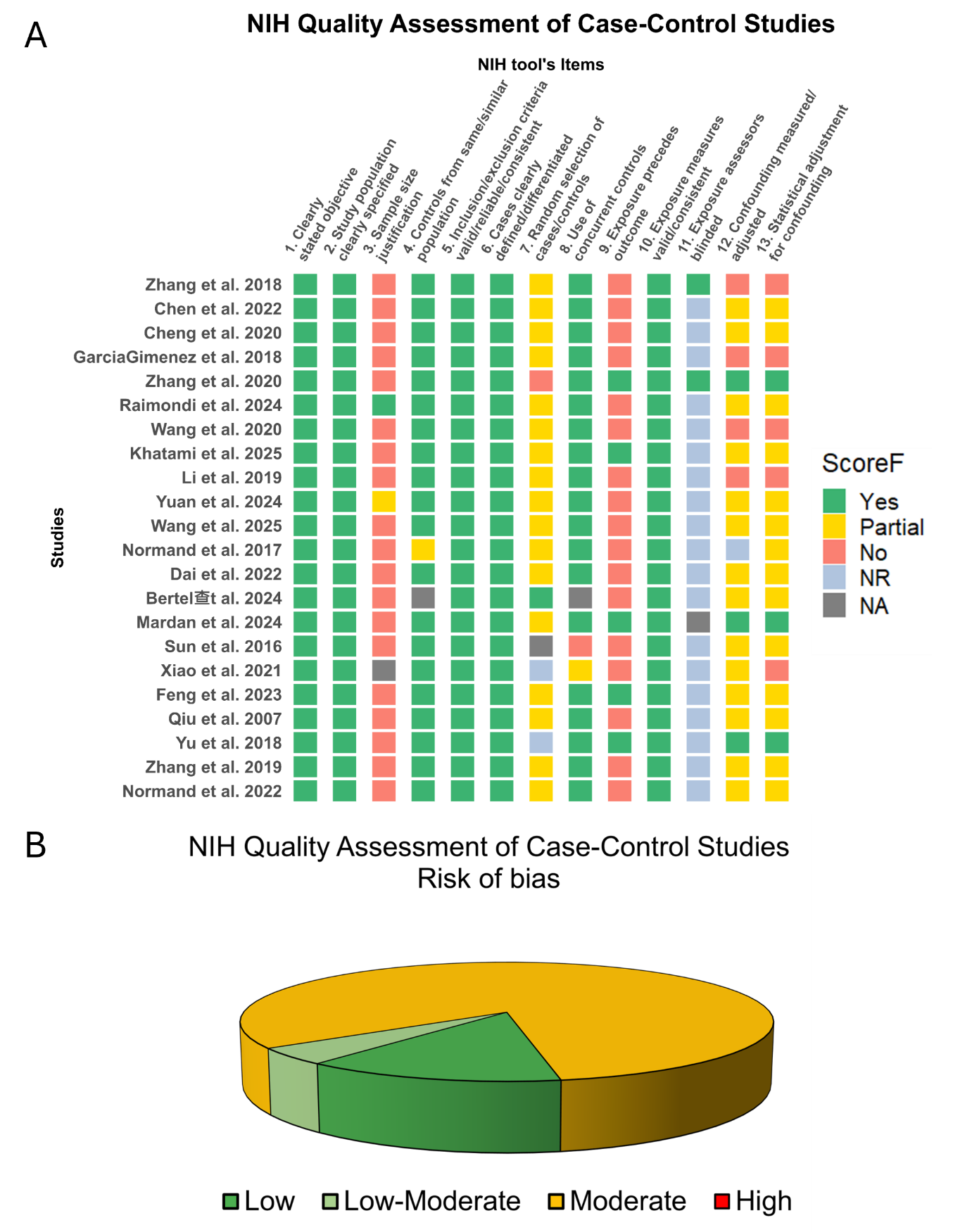


**Supplementary Figure 1.** (**A**), Traffic light heatmap representing the signaling of risk of bias for each included study, across the 13 items of the NIH Quality Assessment Tool for Case-Control Studies. Green (Yes) = low risk, Yellow (Partial) = unclear/moderate risk, Red (No) = high risk, Blue (NR) = not reported, Grey (NA) = not applicable. (**B)**, Pie chart summarizes the overall risk of bias grades assigned to each study based on the collective evaluation across domains, according to the NIH tool (Low, Low-Moderate, Moderate, High risk of bias). Most included studies were evaluated as having moderate risk of bias.

REFERENCE:

National Heart, Lung, and Blood Institute (2021). Study quality assessment tools: quality assessment of case-control studies. Available online at: https://www.nhlbi.nih. gov/health-topics/study-quality-assessment-tools.
